# Advanced retinal disease detection from OCT images using a hybrid squeeze and excitation enhanced model

**DOI:** 10.1371/journal.pone.0318657

**Published:** 2025-02-07

**Authors:** Gülcan Gencer, Kerem Gencer

**Affiliations:** 1 Department of Biostatistics and Medical Informatics, Faculty of Medicine, Afyonkarahisar Health Sciences University, Afyonkarahisar, Turkey; 2 Department of Computer Engineering, Faculty of Engineering, Afyon Kocatepe University, Afyonkarahisar, Turkey; Akita University: Akita Daigaku, JAPAN

## Abstract

**Background:**

Retinal problems are critical because they can cause severe vision loss if not treated. Traditional methods for diagnosing retinal disorders often rely heavily on manual interpretation of optical coherence tomography (OCT) images, which can be time-consuming and dependent on the expertise of ophthalmologists. This leads to challenges in early diagnosis, especially as retinal diseases like diabetic macular edema (DME), Drusen, and Choroidal neovascularization (CNV) become more prevalent. OCT helps ophthalmologists diagnose patients more accurately by allowing for early detection. This paper offers a hybrid SE (Squeeze-and-Excitation)-Enhanced Hybrid Model for detecting retinal disorders from OCT images, including DME, Drusen, and CNV, using artificial intelligence and deep learning.

**Methods:**

The model integrates SE blocks with EfficientNetB0 and Xception architectures, which provide high success in image classification tasks. EfficientNetB0 achieves high accuracy with fewer parameters through model scaling strategies, while Xception offers powerful feature extraction using deep separable convolutions. The combination of these architectures enhances both the efficiency and classification performance of the model, enabling more accurate detection of retinal disorders from OCT images. Additionally, SE blocks increase the representational ability of the network by adaptively recalibrating per-channel feature responses.

**Results:**

The combined features from EfficientNetB0 and Xception are processed via fully connected layers and categorized using the Softmax algorithm. The methodology was tested on UCSD and Duke’s OCT datasets and produced excellent results. The proposed SE-Improved Hybrid Model outperformed the current best-known approaches, with accuracy rates of 99.58% on the UCSD dataset and 99.18% on the Duke dataset.

**Conclusion:**

These findings emphasize the model’s ability to effectively diagnose retinal disorders using OCT images and indicate substantial promise for the development of computer-aided diagnostic tools in the field of ophthalmology.

## Introduction

Diseases of the retina are conditions that seriously jeopardize people’s eyesight and have a direct impact on quality of life. The retina is an essential part of the human eye that is made up of vision cells. It is responsible for processing visual information. The macula, which is required for central vision, is located inside the retinal layer. Retinal damage, particularly in the macular region, can result in a substantial loss of vision [1]. As a result, early identification of retinal anomalies is crucial for facilitating prompt medical treatments as well as reducing vision loss [2]. Among the most prevalent retinal disorders are diabetic macular edema (DME) and age-related macular degeneration (AMD). There are two types of AMD: wet AMD (choroidal neovascularization, or CNV) and dry AMD (drusen), which is the primary cause of blindness in people over 65 [3]. About 25% of diabetic people get DME, which is brought on by fluid buildup in the retina as a result of diabetes problems. These disorders have the potential to permanently impair eyesight if they are not treated in a timely manner. As a result, the development of automated diagnostic systems has become essential for efficient treatment planning, as such systems can reduce the burden on clinicians and improve early detection rates [4].

The detection and treatment of retinal disorders have been completely transformed by advances in medical imaging technologies. A non-invasive imaging technique that produces high-resolution cross-sectional images of the retina is optical coherence tomography. OCT imaging allows for detailed visualization of retinal layers, providing critical insights into retinal health and enabling early diagnosis of conditions like AMD and DME. However, interp images can be time-consuming and requires specialized expertise. To address these challenges, artificial intelligence (AI) and deep learning (DL) methods offer promising solutions for enhancing OCT image analysis and increasing diagnostic accuracy [5]. DL models like as Convolutional Neural Networks (CNNs) have shown impressive results in a number of applications, such as brain tumor and skin cancer detection [6,7]. CNNs have proven useful in ophthalmology for identifying glaucoma from fundus images and segmenting retinal arteries [8]. In the study by Gencer et al. (2024) [9], a comparative analysis of deep learning models was performed in the classification of OCT images. The study stated that the ResNet-101 model provided the highest accuracy and specificity compared to other models. In addition, this study by Gencer and Gencer (2024) [10] emphasizes the success of deep learning models in medical image classification. These AI-powered methods improve clinical decision-making and boost diagnostic precision.

This study proposes a novel SE-Enhanced Hybrid Model for the classification of retinal diseases using OCT images. The model leverages Squeeze-and-Excitation (SE) blocks to adaptively recalibrate feature responses on a per-channel basis, thereby increasing the representational capacity of the network. Additionally, by combining the EfficientNetB0 and Xception architectures, the model benefits from the strengths of both frameworks in feature extraction and classification accuracy. The main contributions of this study are as follows:

- Proposing a novel SE-Enhanced Hybrid Model that integrates EfficientNetB0 and Xception architectures with SE blocks for enhanced feature extraction and classification performance.
- Conducting a comparative analysis that demonstrates the superior accuracy and robustness of the proposed model in classifying retinal diseases such as DME, CNV, and drusen.
- Providing a comprehensive evaluation of the model using two publicly available OCT datasets (UCSD and Duke), highlighting its generalizability and applicability in clinical settings.
- Addressing the computational efficiency of the model, making it a feasible solution for real-time diagnostic applications in ophthalmology.

The remaining sections of this article are organized as follows. First, a comprehensive analysis of related research is provided. Next, the proposed methodology is detailed, covering CNN, SE blocks, the SE-Enhanced Hybrid model architecture, and the datasets used. The subsequent section presents the experimental results, followed by a discussion of the study’s limitations. Finally, the article concludes with a summary of the findings and suggestions for future research directions.

## Related work

With the growth of computing systems in ophthalmology and the advancement of imaging technology, the analysis and categorization of OCT images is becoming more and more popular. Because of new technologies and increased processing capacity, artificial intelligence-supported decision support systems are being used in this industry more and more. This section summarizes current research with OCT datasets that are comparable to the study’s.

Using the Inception V3 architecture that has been pre-trained on the ImageNet dataset, Kermany et al. (2018) [11] created a transfer learning system for medical image analysis. When evaluated on OCT images, this technique effectively identified retinal diseases. Similar to this, Silva et al. (2023) [12] compares four distinct ensemble CNN models with current CNN architectures while utilizing transfer learning.

Kim and Tran (2020) [13] examined and assessed for the classification of OCT images. They also used an ensemble learning approach based on several ResNet152 architectures to achieve great performance, and they employed Fully Convolutional Networks (FCN) to remove noise. DenseNet was utilized by Rastogi et al. (2019) [14] to identify retinal abnormalities from OCT images, and they emphasized how successful it was in comparison to conventional CNNs.

Unlike typical CNNs, a capsule network application was developed to extract spatial information from images [15]. A deep learning-based approach that uses OCT images to diagnose macular disorders has been suggested [16]. The suggested model achieves great accuracy and performance by utilizing the VGG-16, VGG-19, and ResNet architectures. For automatic OCT image classification, we used a deep transfer learning approach based on VGG-16. Comparing CNN and transfer learning models for classifying diabetic macular edema, drusen, and choroidal neovascularization yielded an accuracy of up to 99.38% [17].

A layer-guided convolutional neural network (LGCNN) was proposed to classify retinal diseases using retinal layer segmentation [18]. Deep learning techniques are also being used to minimize noise in OCT images, which aids in the more accurate diagnosis and classification of retinal disorders. Mehdizadeh et al. (2021) [19] provide a technique to denoise OCT images and improve perceptual acuity that combines deep feature loss with a VGG network. Hu et al. (2023) [20] present a selective denoising technique that increases classification accuracy by first classifying CNN-classified images that need noise reduction before utilizing the BM3D algorithm. The semi-supervised Capsule cGAN approach, which mixes supervised and unsupervised losses for speckle noise reduction, is proposed by Wang et al. (2021) [21].

A generative adversarial network (GAN) is used by Hasan et al. (2021) [22] to examine OCT images. A generator that creates denoised images from noisy inputs and a discriminator that discerns between actual and denoised images are components of the Gan architecture. Better performance measurements, such peak signal-to-noise ratio (PSNR), demonstrate how the GAN-based approach works better than conventional denoising approaches like wavelet-transform, bilateral, non-local means (NLM), and BM3D. Using a variety of datasets, Bogacki and Dziech (2023) [23] provide a novel deep learning technique for testing OCT images. Through the use of BM3D filtering, pairs of clean and noisy images are used to train a deep learning model. Several quantitative criteria have demonstrated the huge improvement in image quality that denoising brings.

CNN models like ResNet50 and Xception were used to identify disorders in retinal OCT images. They discovered that MobileNetV2 was the most effective [24]. Zhu et al. (2024) [25] created nn-MobileNet, a CNN model that can be utilized on mobile devices, by reevaluating the CNN designs used for the diagnosis of retinal disorders. Using four distinct public datasets, this model has proven to perform better on a variety of tasks, such as the categorization of diabetic macular edema, fundus multimorbidity identification, and diabetic retinopathy.

A completely automated technique is presented by Mittal and Bhatnagar (2022) [26] to use OCT images to diagnose disorders like DME. High accuracy rates were obtained by employing SVM-based classifier with HOG descriptors.

A framework for the automated identification of the retinal pigment layer using spectral domain OCT images was proposed by Naz et al. (2016) [27]. Khalid et al. (2016) [28] developed a classification system that employs numerous fitting curve procedures, including noise reduction approaches, together with intensity-based thresholding for age-related drusen identification. In their study, Salaheldin et al. [29] propose a deep learning-based model for automatic detection and grading of papilledema from OCT images. The model aims to aid clinical diagnosis and management by providing an accurate and efficient way to determine and assess the severity of papilledema.

This study by Salaheldin et al. (2024) [30] presents a hybrid model that utilizes artificial intelligence techniques for the detection of retinal disorders using OCT images. The model combines various machine learning approaches to increase accuracy and diagnostic confidence in identifying retinal conditions. Kayadibi and Güraksın (2023) [31] developed a CNN-based stacking ensemble learning (EL) method to detect common retinal diseases such as diabetic macular edema, choroidal neovascularization, and drusen from OCT images. The study performed high accuracy classification using homogeneous and heterogeneous EL methods and achieved over 99% success on Duke and UCSD datasets. This method contributes to early diagnosis by increasing the accuracy of computer-aided diagnostic tools.

A review of the literature reveals that classical machine learning classifiers dominated the research of OCT datasets such as Duke [3,27,32]. The other dataset, originally provided by Kermany et al. [11], is commonly used to evaluate deep learning algorithms, notably CNN architectures. This paper proposes a hybrid SE-Enhanced Hybrid Model architecture for detecting retinal illness from OCT images, which combines SE blocks for improved feature representation with EfficientNetB0 and Xception for effective feature extraction.

## Materials and methods

### Datasets

This work used two publicly available OCT datasets, the UCSD and Duke datasets, as shown in Table 1. These datasets include photographs of both normal retinas and retinas affected by disorders.

**Table 1:** Details of the datasets.

Category	Train	Test	Image Size	Source
NORMAL	26315	243	224 × 224	Shiley Eye Institute
CNV	37205	243	224 × 224	California Retinal Research
DRUSEN	8616	243	224 × 224	Medical Center Oph. Associates
DME	11348	243	224 × 224	Shanghai First Peop. Hospital
NORMAL	142	51	224 × 224	Duke University
AMD	98	41	224 × 224	Duke University
DME	126	30	224 × 224	Duke University

- The first dataset is presented by Srinivasan et al. [33]. This collection provides OCT images used to diagnose and classify retinal disorders, including NORMAL, AMD, and DME.
- The second dataset was given by Kermany et al. [11]. This dataset includes 84,495 OCT images classified into four categories: NORMAL, CNV, DME, and DRUSEN.

### Description

In this paper, a hybrid SE-Enhanced Hybrid Model architecture is suggested for detecting prevalent retinal disorders using OCT images. A hybrid deep learning model combining two powerful convolutional neural network (CNN) architectures was implemented. These are EfficientNetB0 and Xception. This hybrid model also included Compression and Excitation (SE) blocks to improve feature extraction. The purpose of this model is to categorize images in the OCT dataset. SE blocks were used to recalibrate feature responses on a channel-by-channel basis by explicitly modeling interdependencies between channels. This helped improve the representativeness of the network. The model combined the outputs of EfficientNetB0 and Xception architectures, both enhanced with SE blocks, to create a robust feature extraction mechanism. It was passed through fully connected layers to perform classification.

**Fig 1:**
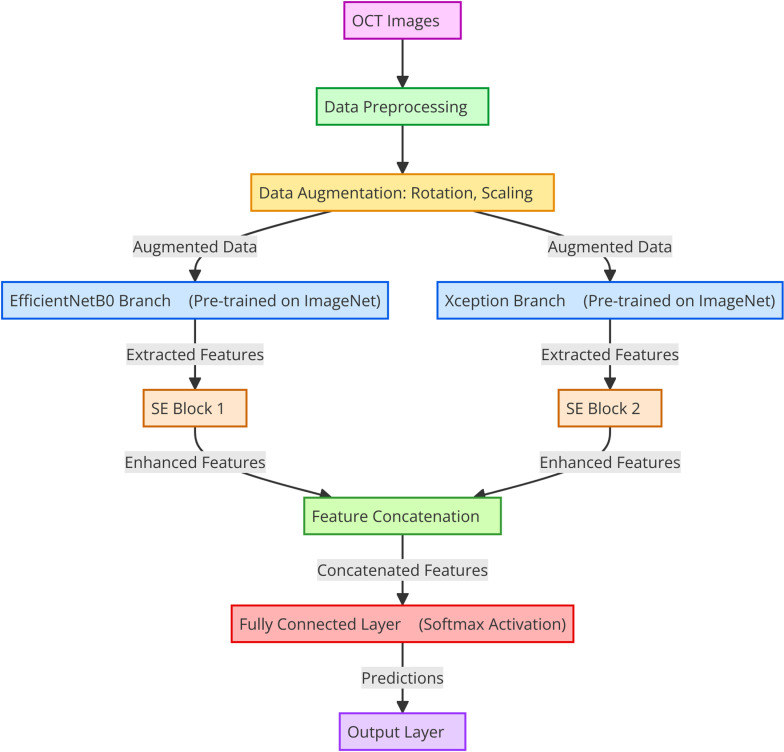
The proposed approach in OCT.

Images were resized to 224 × 224 pixels and rescaled to the [0, 1] pixel density range. Data augmentation techniques such as random rotations, shifts, shear transformations, zooms, and horizontal flips were applied to improve the training data and prevent overfitting. The model was trained using Adam optimizer with a learning rate of 0.0001. Model checkpoints, reducing the learning rate at the plateau, and stopping callbacks early were used to improve training efficiency and prevent overfitting. The technique illustrated in Fig 1 involves a preprocessing step to ensure that the set of data is scaled to the appropriate input sizes for previously trained CNN models.

### Convolutional neural network

**Fig 2:**
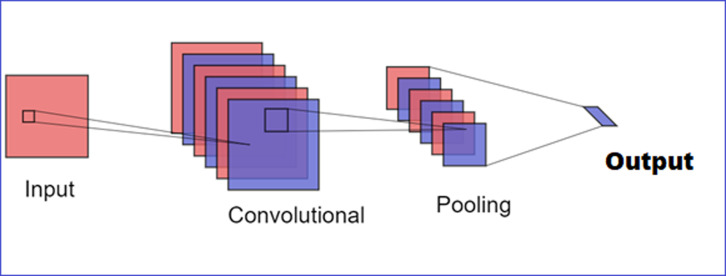
Visual representation of CNN architecture.

Convolutional Neural Networks (CNNs) are sophisticated neural networks that imitate the visual processing systems of living things in order to interpret and evaluate visual input. CNNs are a subset of deep learning that have shown remarkable performance in a wide range of data-driven applications because of their multilayer structure, which makes it possible to extract hierarchical characteristics from input images. Fig 2 shows an example of a convolutional neural network design. CNNs were brought to the forefront in computer vision research by Krizhevsky and others. When they won the ImageNet Large Scale Visual Recognition Competition (ILSVRC) with their AlexNet architecture. This achievement, highlighting the ability of CNNs for unsupervised feature extraction and setting a new standard for image classification tasks [34]. A typical CNN architecture consists of several types of layers, including convolutional layers, pooling layers, and fully connected layers:

Convolutional Layers apply a series of filters (kernels) to the input image, creating feature maps that capture various aspects of the image, such as edges, textures, and patterns. Mathematically, the convolution process for an input image *I* and *K* can be represented as:


$$\left(I*K\right)\left(i,j\right)=\underset{m}{\sum }\underset{n}{\sum }I\left(i+m,j+n\right)K\left(m,n\right)$$ (1)

where (m, n) are the filter’s coordinates and (i, j) are the coordinates of the input image. Through this procedure, the network may directly learn the spatial hierarchies of features from raw pixel data [35]. An activation function, such the Rectified Linear Unit (ReLU), is applied after every convolution layer. The definition of the ReLU function is:


$$ReLU\left(x\right)=max\left(0,x\right)$$ (2)

ReLU introduces nonlinearity into the model, allowing it to learn more complex patterns and interactions within the data [36].

These layers perform subsampling operations, reducing the spatial dimensions of feature maps while preserving the most critical information. A common pooling process is maximum pooling, defined as:


$$P\left(i,j\right)=max_{m,n}\left(I\left(i+m,j+n\right)\right)$$ (3)

where (i + m,j + n) denotes the local region where the most processing is done, and P(i,j) is the aggregated result. This makes the model more resilient to geographical fluctuations in the input data and computationally efficient [37]. Fully connected layers in a CNN integrate the characteristics that the convolutional layers retrieved to arrive at a final classification conclusion. These layers function similarly to those found in conventional neural networks, linking each neuron in one layer to each subsequent layer’s neuron. They are depicted as follows:


$$y=f\left(Wx+b\right)$$ (4)

where x is the input, y is the output, b is the bias, and f is an activation function. W is the weight matrix [34].

### Transfer learning

A pre-trained model created for one job is utilized as the basis for a new model on a different task, a process known as transfer learning in machine learning. By using the information acquired from the first activity, this method enhances performance and shortens the training period for the subsequent task [38]. Transfer learning in image classification refers to the use of models that have already been pre-trained on huge datasets, like ImageNet, which has millions of classified photos organized into thousands of categories. These pre-trained models are helpful for a variety of image identification tasks because they have learnt to extract generic characteristics from images, such as edges, textures, and forms [39]. Pre-trained models like Xception, EfficientNetB0, AlexNet, VGG16, InceptionV3, and ResNet are frequently used in image categorization. These models have demonstrated strong performance in a range of image identification tasks after being trained on extensive image datasets [40–42]. By keeping the convolution layers intact and eliminating the final classification layer, the pre-trained model is utilized as a feature extractor. From the input photos, these layers extract characteristics that are relevant. A new classifier trained on the target dataset may then be fed the extracted features. On the target dataset, the pre-trained model is occasionally further refined. This involves retraining some or all of the layers of the pre-trained model on new data. Fine-tuning allows the model to adapt previously learned features to the specific characteristics of the new data set. Features extracted from the pre-trained model are fed into a new classifier, usually a fully connected neural network or a support vector machine (SVM) trained to perform the final classification task.

The Xception model, which is a Convolutional Neural Network (CNN) structure and an enhanced version of Google’s Inception model, is one of these models. “Extreme Inception” is what Xception stands for, and its main objective is to improve deep learning model performance and accuracy [43].In his paper Sadik et al. (2023 [44]), investigated the use of transfer learning and the Xception model for the diagnosis of skin disorders. Hirahara (2019) [45] conducted a preliminary assessment of the Xception model’s application to transfer learning in the creation of the CADe system, which is used to identify brain tumors. Mukhlif and Al-Khateeb (2023) [46] talked about the use of the Xception model for breast cancer image classification as well as novel transfer learning strategies. This study used several well-known pre-trained CNN architectures as part of the SE-Enhanced Hybrid Model methodology to classify retinal diseases from OCT images. Selected architectures include EfficientNetB0 and Xception. Each of these models has demonstrated high performance on a variety of image classification challenges, making them suitable candidates for transfer learning in medical image analysis. EfficientNetB0 achieves high efficiency using model scaling strategies and delivers high accuracy with a low number of parameters. Xception, on the other hand, performs powerful feature extraction using deep discrete convolutions and exhibits high classification performance. SE blocks increase the representativeness of the network and improve overall performance by adaptively recalibrating feature responses on a channel-by-channel basis. This hybrid model has been utilized as a potent tool for detecting retinal disorders from OCT images, and its usefulness has been tested using OCT datasets. The use of SE blocks and specific CNN topologies improves classification accuracy, allowing for more trustworthy outcomes in medical diagnosis processes. Table 2 summarizes the characteristics of the CNN architectures employed in this investigation.

**Table 2:** Features of CNN architectures.

Model	Number of Layers	Number of Parameters	Input Size (px)	Features
EfficientNetB0	237	5.3 M	224 × 224	Scaling, MBConv Blocks, Swish Activation, Squeeze-and-Excitation Blocks
Xception	71	22.9 M	224 × 224	Depthwise Separable Convolutions, Residual Connections, Extreme Inception

### Preprocessing

The proposed method’s preprocessing phase ensures that the raw data set is scaled to fit the input parameter sizes of previously trained CNN architectures. The dataset’s training and testing images for EfficientNetB0 and Xception were trimmed to 224 ×  224 pixels to fit the input dimensions of these CNN architectures. By making sure the images complied with CNN architectures’ input specifications, our procedure readied them for both training and testing. The pixel values of each image were divided by 255 to rescale them to the [0, 1] pixel intensity range. Because normalization provided constant input value ranges, it helped stabilize the training process. To suit the input dimensions required by the CNN models employed in this work, all photos were scaled to 224 ×  224 pixels. To reduce overfitting and boost model resilience, training images are subjected to a variety of data augmentation strategies. These methods included applying shift transformations to photos, randomly zooming in on images, randomly flipping images horizontally, and randomly rotating images to a certain extent.

### Details of the proposed SE-enhanced hybrid architecture and classification model

In this study, SE-Enhanced Hybrid Model methodology is proposed to classify retinal diseases from OCT images. The core of this model is based on a custom CNN that integrates SE blocks to improve feature extraction. SE Blocks are a special block structure used to increase the feature extraction power of the network by recalibrating the importance of each channel. This structure increases the representativeness of the features by generating adaptive weights for each channel. A strong classification performance was achieved by using EfficientNetB0 and Xception architectures in the model. EfficientNetB0 and Xception models were used to extract features from OCT images. EfficientNetB0 provides high accuracy with high efficiency and low number of parameters by using model scaling strategies. Xception, on the other hand, performs powerful feature extraction using deep discrete convolutions. SE (Squeeze-and-Excitation) blocks are integrated after each convolutional layer to adaptively recalibrate feature responses on a per-channel basis [47]. This is accomplished through a two-step process: squeezing and stimulation. Mathematically, the SE block is defined as:


$$s=\sigma \left(W_{2}.\delta \left(W_{1}.z\right)\right)$$ (5)

where *z* is the input, $W_{1}$ and $W_{2}$ are the weights, *δ* is the ReLU activation, and *σ* is the σsigmoid activation^44^. After convolution and SE blocks, the extracted features are smoothed and passed through a dense layer with 128 neurons followed by a dropout layer to avoid overfitting. These combined features are passed through the Dense layer and the Dropout layer. In the final stage, retinal diseases are classified using a Softmax classifier. Softmax classifier is an activation function used to classify multiple classes. It converts the outputs into probability values for each class and ensures that the class with the highest probability is selected [48].The performance of the model is evaluated with metrics such as accuracy, precision, recall and F1 score.

The SE-Hybrid architecture is trained using the Adam optimizer with a learning rate of 0.0001. The model was trained for 30 epochs with a batch size of 32. During training, data augmentation techniques such as rotation, width and height shifting, cropping, zooming, and panning are applied to increase the robustness of the model and prevent overfitting. The SE-Enhanced Hybrid Model provides a robust framework for classifying retinal diseases from OCT images. By combining the representative power of SE blocks and the feature extraction efficiency of CNNs, significant improvements in classification performance have been achieved. This model has been tested on OCT datasets, demonstrating its effectiveness in detecting retinal diseases.

## Experiments and results

This paper proposes a hybrid SE-Enhanced Hybrid architecture for detecting retinal disorders utilizing retinal images. Initially, the model was created with pre-trained CNN architectures such as EfficientNetB0 and Xception. These models stand out in medical image analysis with their high accuracy and efficiency. EfficientNetB0 and Xception were used to extract features from images. EfficientNetB0 offers scalability and high accuracy, while Xception provides powerful feature extraction using deep discrete convolutions. SE (Squeeze-and-Excitation) blocks are integrated after each convolutional layer to adaptively recalibrate feature responses on a per-channel basis. This comprehensive evaluation highlights the effectiveness of the SE-Enhanced Hybrid Model in accurately detecting retinal diseases from images.

### Experimental preparation

All experiments on the SE-Enhanced Hybrid architecture suggested in this paper, as well as pre-trained CNN architectures, were carried out in Python with the TensorFlow and Keras libraries. The experiments were performed on a computer with the following specifications: Operating System, Windows 10, Processor:,Intel(R) Core i7 2.6 GHz, Graphics Card, Nvidia GTX 1650Ti, Software Environment, Python 3.8, TensorFlow 2.x, Keras 2.x.

### Efficiency and resource utilization

Table 3 presents a comparison of model training and inference efficiencies between the UCSD and Duke datasets, highlighting key metrics such as training time, inference time, computational FLOPs, and resource utilization (GPU and CPU memory).

**Table 3:** Comparison of Performance and Resource Usage of UCSD and DUKE Models.

Methods	Description	Metrics	UCSD Dataset	DUKE Dataset
			Value	Value
SE-EfficientNetB0	Model training time	Duration	63.31 minutes	4.20 minutes
	Inference time per image	Duration	13.87 ms	169.36 ms/image
	Total FLOPs (billion floating-point operations)	FLOPs	0.80 GFLOPs	0.81 GFLOPs
	GPU memory used during training	Memory	14225 MB	52.16 MB
	CPU memory used during training	Memory	10126 MB	5399.81 MB
SE-Xception	Model training time	Duration	62.21 minutes	4.52 minutes
	Inference time per image	Duration	8.09 ms/image	83.47 ms/image
	Total FLOPs (billion floating-point operations)	FLOPs	9.14 GFLOPs	0.81 GFLOPs
	GPU memory used during training	Memory	12921 MB	107.29 MB
	CPU memory used during training	Memory	15997 MB	6719.31 MB
SE-Hybrid	Model training time	Duration	73.30 minutes	8.73 minutes
	Inference time per image	Duration	18.69 ms	43.65 ms/image
	Total FLOPs (billion floating-point operations)	FLOPs	9.14 GFLOPs	9.94 GFLOPs
	GPU memory used during training	Memory	12917 MB	359.01 MB
	CPU memory used during training	Memory	13910 MB	7524.41 MB

### Hyperparameters and training

The hybrid model was created by adding SE blocks to the outputs of EfficientNetB0 and Xception architectures. SE blocks increased the representativeness of the network by adaptively recalibrating feature responses on a channel-by-channel basis. The outputs of EfficientNetB0 and Xception models were combined with global average pooling layers, and then final classification was performed with fully connected (FC) layers. In the last layer of the model, classification was performed using the Softmax activation function. For training the model, data was prepared using ImageDataGenerator and divided into training, validation and test sets. The model was trained for 30 epochs and during the training process. The decision to limit training to 30 epochs was informed by preliminary experiments in which we observed the model’s performance trends, including training and validation accuracy and loss metrics. In these experiments, the model achieved a high accuracy level early in training, and additional epochs did not yield significant performance gains, instead showing signs of potential overfitting. To prevent overfitting, we also employed early stopping, which further reinforced the decision to limit training to this duration, as the model often converged or reached optimal performance within the chosen range. Given the high parameter count and volume of data, we aimed to balance computational efficiency with model generalization, and 30 epochs were determined to be sufficient to reach a robust performance level. Additionally, to strengthen the evaluation of generalization capability, we included the Matthews Correlation Coefficient (MCC) metric in our analysis, alongside accuracy, precision, recall, and specificity, to provide a more comprehensive view of the model’s performance.

The optimization technique used in this study was Adaptive Moment Estimation (Adam), selected to optimize model performance due to its effectiveness in handling sparse gradients. Adam was configured with a learning rate of 0.0001, based on preliminary testing to balance convergence speed and accuracy. Table 4 gives more information on these hyperparameters. The training approach also included tactics such as ReduceLROnPlateau, Early Stopping, and model checkpointing. The effectiveness of the model’s categorization was evaluated using cross-entropy loss. The cross entropy loss criteria in this study, which is used with Softmax in the last layer of the model, has the following mathematical expression:

**Table 4:** Specifics of the architecture-related hyperparameters.

Parameter	Description	Value/type
Loss Criterion	Defines the function used to optimize classification accuracy by penalizing incorrect predictions.	Categorical Cross-entropy
Optimizer	Algorithm used to adjust model weights, improving accuracy while minimizing loss.	Adam
Learning Rate	Controls the step size during weight updates to find the optimal model parameters.	0.0001
Batch Size	Number of samples processed before updating model weights.	32
Epochs	Number of complete passes through the training dataset during model training.	30
Network Output Classes	Categories for final classification predictions, representing types of retinal disorders.	CNV, DME, Drusen, Normal


$$H\left(p,q\right)=-\underset{x}{\sum }p\left(x\right)logq\left(x\right)$$ (6)

In this case, q stands for the Softmax output, *z* for the number of classes, and *P* for the output of the categorical classes. Non-linear outputs can be generated using CNN training functions [49].

### Performance metrics

The efficiency of the models used in this study was evaluated using a commonly used confusion matrix. Performance measures including precision (Equation 7), sensitivity (Equation 8), specificity (Equation 9), accuracy (Equation 10), and F1 score (Equation 11) were computed using this matrix. To evaluate the performance of the classification models, Matthews Correlation Coefficient (MCC) statistical measures is provided in Equations 12. MCC is a balanced measure used in binary classification problems, evaluating the overall performance of the classifier. It provides reliable results even when there is an imbalance between positive and negative classes [50].


$$Presicion=\frac{TP}{TP+FP}$$ (7)

$$Recall\left(Sensitivity\right)=\frac{TP}{TP+FN}$$ (8)

$$Specifity=\frac{TN}{TN+FP}$$ (9)

$$Accuracy=\frac{TP+TN}{TP+TN+FP+FN}$$ (10)

$$F1Score=2*\frac{Precision\times Recall}{Precision+Recall}$$ (11)

$$MCC=\frac{\left(TP\times TN\right)-\left(FP\times FN\right)}{\sqrt{\left(TP+FP\right)\left(TP+FN\right)\left(TN+FP\right)\left(TN+FN\right)}}$$ (12)

True positives (TP), true negatives (TN), false positives (FP), and false negatives (FN) are the terms used in these equations. The system’s F1 score, accuracy, sensitivity, specificity, and precision were calculated using these data. Specificity guarantees the genuine negative rate, whereas accuracy shows how closely measurement findings match the true value. Another important criterion in performance evaluation is the ROC (Receiver Operating Characteristic) curve. The ROC curve enables comprehensive reporting of sensitivity and specificity rates.

In this study, the performance of the SE-Enhanced Hybrid architecture and other pre-trained models was evaluated using the aforementioned metrics. Confusion matrix was calculated for each model and accuracy, sensitivity, specificity, precision and F1 score were calculated using TP, TN, FP and FN values. Additionally, ROC curves were plotted for each class.

### EfficientNetB0, Xception versus SE-enhanced hybrid on OCT dataset

**Fig 3:**
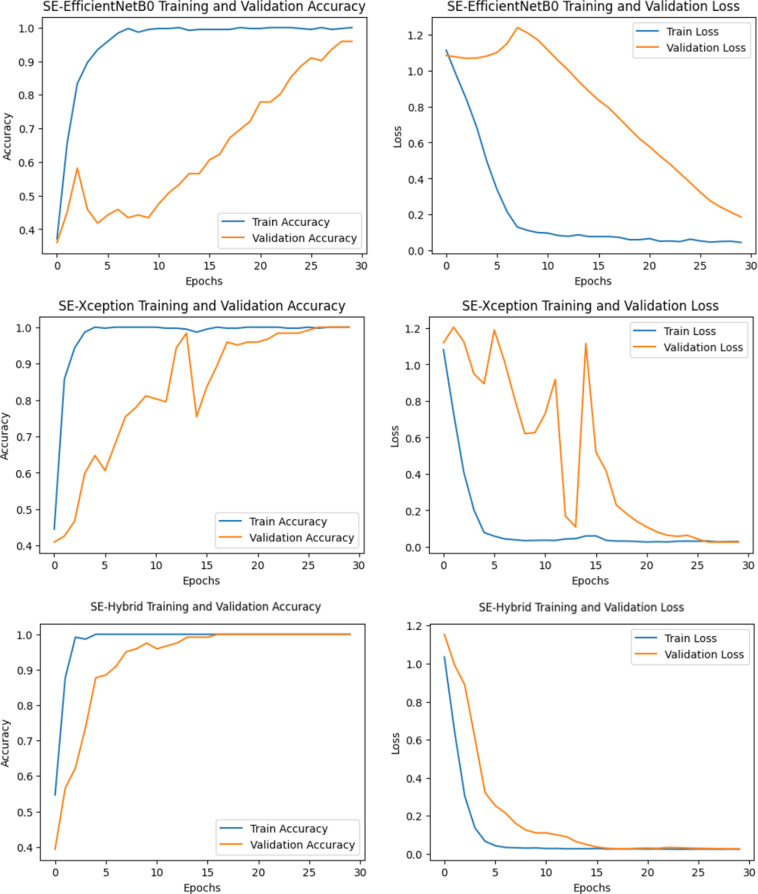
Training and Validation Performance of SE-EfficientNetB0, SE-Xception and SE-Hybrid Models on the Duke Dataset: Comparison of Accuracy and Loss Metrics Across Epochs.

**Fig 4:**
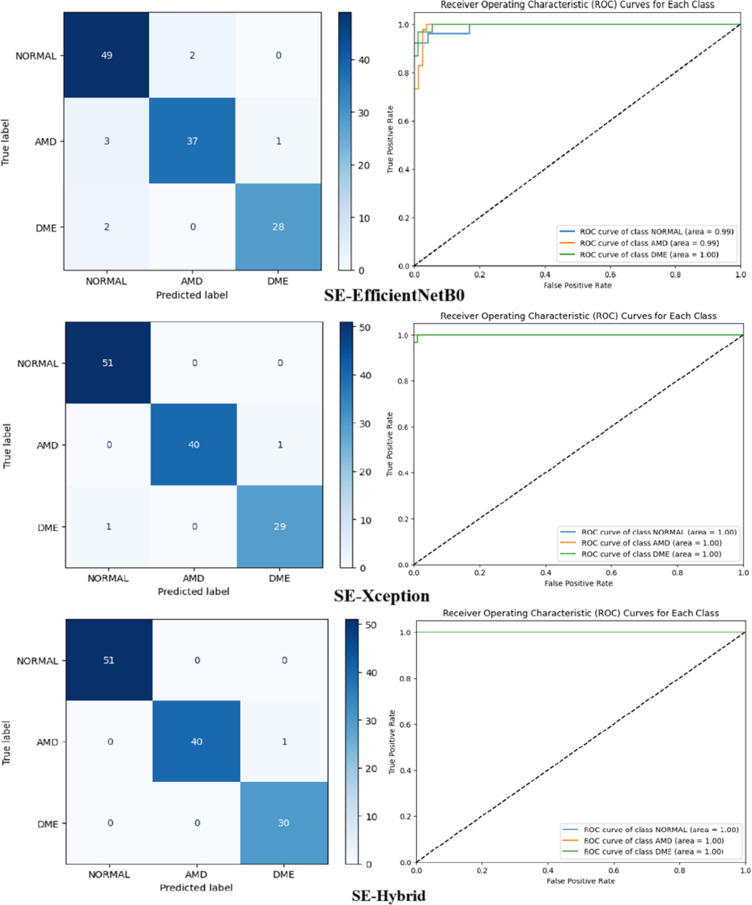
Comparative Analysis of Confusion Matrices and ROC Curves for SE-EfficientNetB0, SE-Xception and SE-Hybrid Models on the Duke Dataset.

**Fig 5:**
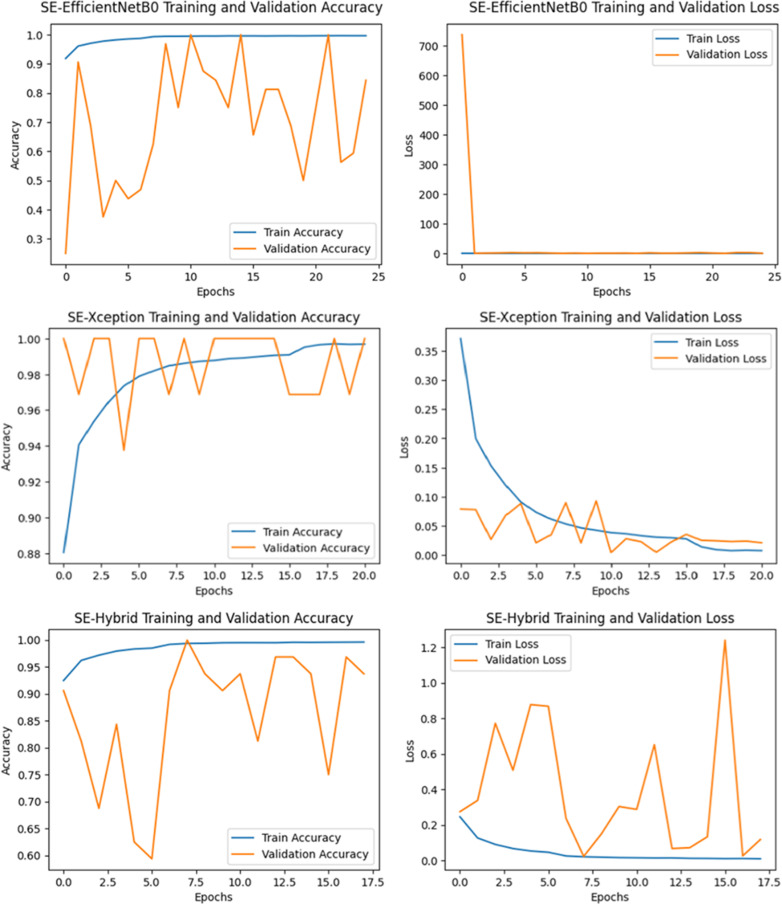
Training and Validation Performance of SE-EfficientNetB0, SE-Xception and SE-Hybrid Models on the UCSD Dataset: Comparison of Accuracy and Loss Metrics Across Epochs.

**Fig 6:**
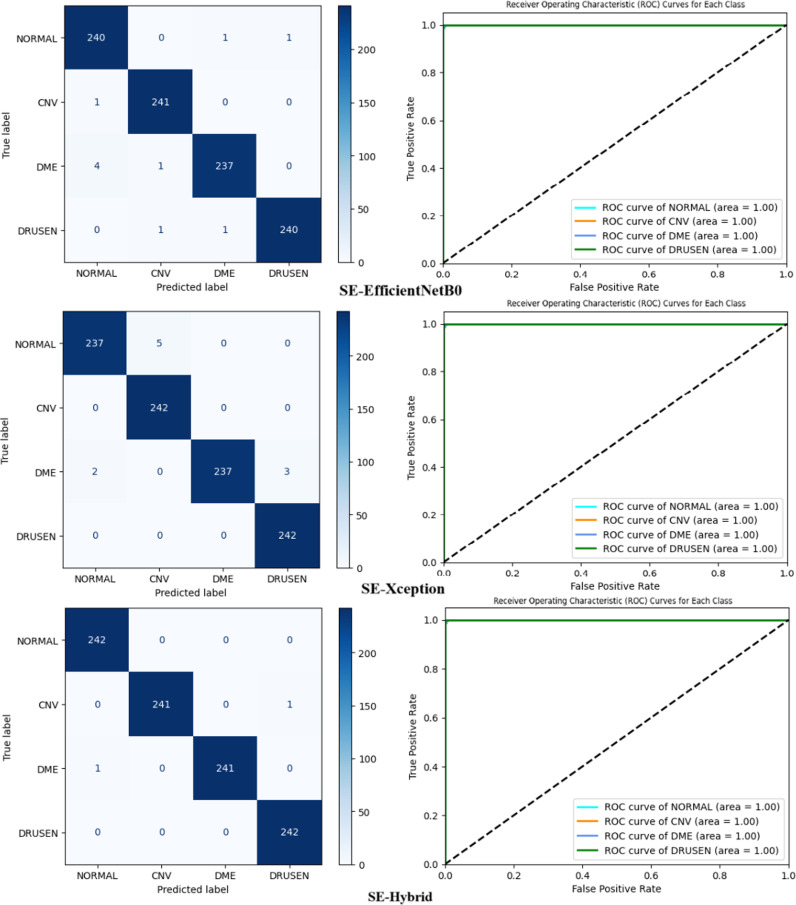
Comparative Analysis of Confusion Matrices and ROC Curves for SE-EfficientNetB0, SE-Xception and SE-Hybrid Models on the UCSD Dataset.

In the “Details of the proposed SE-Enhanced hybrid architecture and classification model” section, a hybrid architecture based on the ability to detect retinal diseases is proposed and detailed. Tables 5 and 6 show performance measurements of SE-Enhanced Hybrid against EfficientNetB0 and Xception for the Duke and UCSD datasets, respectively (best results are underlined in bold). In terms of all performance parameters, the SE-Enhanced Hybrid fared better than the EfficientNetB0 and Xception. This section summarizes the performance comparisons between SE-Enhanced Hybrid, EfficientNetB0 and Xception CNN architectures using ROC curves, confusion matrices and training and loss graphs. For the first data set, they are displayed in Figs 3 and 4). For the second data set, it can be seen in Figs 5 and 6. The best-performing CNN architecture is SE-Enhanced Hybrid, as can be shown in all figures.

**Table 5:** Performance metrics of SE-EfficientNetB0, SE-Xception and SE-Hybrid on first dataset (Duke).

Technique	Accuracy	Recall	Specificity	Precision	F1 score	MCC
SE-EfficientNetB0	0.9344	0.9321	0.9321	0.9405	0.9358	0.8996
SE-Xception	0.9836	0.9807	0.9807	0.9824	0.9815	0.9749
SE-Hybrid	**0.9918**	**0.9918**	**0.9918**	**0.9892**	**0.9904**	**0.9875**

**Table 6:** Performance metrics of SE-EfficientNetB0, SE-Xception and SE-Hybrid second dataset (UCSD).

Technique	Accuracy	Recall	Specificity	Precision	F1 score	MCC
SE-EfficientNetB0	0.9896	0.9896	0.9896	0.9838	0.9896	0.9862
SE-Xception	0.9938	0.9938	0.9938	0.9939	0.9938	0.9917
**SE-Hybrid**	**0.9958**	**0.9958**	**0.9958**	**0.9959**	**0.9958**	**0.9972**

### Comparison with state-of-the-art deep learning based techniques

In this section, we compare the proposed SE-Enhanced Hybrid Model with the state-of-the-art deep learning techniques used for classification. Table 7 provides a summary of these techniques and performance measures. It shows that the proposed SE-Enhanced Hybrid Model significantly outperforms the existing state-of-the-art methods on different datasets: The proposed SE-Enhanced Hybrid Model achieves 99.58% accuracy on the UCSD dataset and 99.18% accuracy on the Duke dataset. These results exceed the highest accuracy rates reported by Kim and Tran (2020) [13] and Naz et al (2016)[27]. Kermany et al. (2018) [11] achieved an accuracy of 96.6% using transfer learning with InceptionNet V3. In contrast, the SE-Enhanced Hybrid Model improves this accuracy by approximately 3%, highlighting the effectiveness of including SE blocks and combining EfficientNetB0 with Xception architectures. Techniques that include image preprocessing and feature extraction, such as Naz et al.‘s (2016) [27] and Khalid et al. (2016) [28] report varying accuracies (98.00% and 92.00%, respectively). The proposed method not only simplifies the process by leveraging advanced deep learning architectures, but also significantly increases the accuracy.Rastogi et al. (2019) [14] used densely connected convolutional neural networks and achieved. The superior accuracy of the proposed SE-Enhanced Hybrid Model demonstrates its improved ability to learn and generalize from images. The integration of Compress and Excitation (SE) blocks increases the representation capacity of the model by improving feature recalibration. This approach, combined with the hybrid architecture of EfficientNetB0 and Xception, contributes to the outstanding performance of the model. The proposed SE-Improved Hybrid Model shows significant improvements in image classification accuracy compared to existing state-of-the-art methods. Effectively combining EfficientNetB0 and Xception architectures with SE blocks, the model leverages advanced feature extraction and recalibration techniques, providing superior performance on both UCSD and Duke datasets.

**Table 7:** Summary of techniques compared to state-of-the-art technologies for OCT classification using different datasets.

Source	Proposed Method	Dataset	Accuracy (%)	Recall (%)	Specificity (%)
Kermany et al. (2018)	Transferring knowledge with InceptionNet V3	UCSD	96.60	97.80	97.40
Kim and Tran (2020)	Using ResNet152 for Ensemble Learning		98.90	98.90	99.60
Rastogi et al. (2019)	Convolution Neural Network with Dense Connectivity		98.00	95.57	99.15
**Proposed Method**	**SE-Enhanced Hybrid**		**99.58**	**99.58**	**99.58**
Naz et al. (2016)	SVM classifiers, RPE extraction, and image pre-processing	DUKE	96.00	–	–
Khalid et al. (2016)	Pre-processing images, RPE extraction, and classifiers based on polyfitted curves		92.00	–	–
**Proposed Method**	**SE-Enhanced Hybrid**		**99.18**	99.18	99.18

## Limitations

Although our study obtained promising results, it has some limitations. First, the datasets used are limited to the UCSD and Duke datasets. It is necessary to test the performance of the model on different data sets other than these data sets and evaluate its generalization ability. Second, the computational resources and time used during training and testing the model can be quite high due to large data sets and complex models. Third, the performance of the model is limited to certain retinal diseases, and its validity for other retinal diseases needs to be investigated. Finally, the applicability of the model in clinical settings and its performance in real-world conditions need to be evaluated. These limitations are important considerations for future studies.

## Conclusion

In recent years, with the advancement of technology, artificial intelligence has achieved great success in the field of medicine and has begun to be widely used. Medical imaging devices also evolve with these technological advances. In clinical settings, specialist physicians can interpret images; However, the duration and accuracy of this process largely depends on the experience of the specialist. As a result, this study proposes employing a hybrid SE-Enhanced Hybrid Model to detect retinal disorders in OCT images. The suggested model combines SE blocks, EfficientNetB0, and Xception architectures. SE blocks improve the network’s representational ability by adaptively adjusting per-channel feature responses. The combined features from EfficientNetB0 and Xception are processed via fully connected layers and categorized using the Softmax algorithm. The methodology was tested on UCSD and Duke’s datasets and produced excellent results. The proposed SE-Improved Hybrid Model outperformed the best available approaches, achieving an accuracy rate of 99.58% on the UCSD dataset and 99.18% on the Duke dataset. As seen in Tables 4 and 5, the proposed method outperformed the previous approaches. Tables 4 and 5 exhibit performance comparisons for the Duke dataset and the UCSD dataset, respectively. The results suggest that the proposed strategy outperforms alternative approaches. Future work may focus on expanding the model’s validation on diverse OCT datasets and investigating its applicability in real-time clinical settings. Additionally, integrating other feature extraction techniques and further optimizing the SE blocks could enhance model performance, making it a robust tool for broader applications in ophthalmology.
